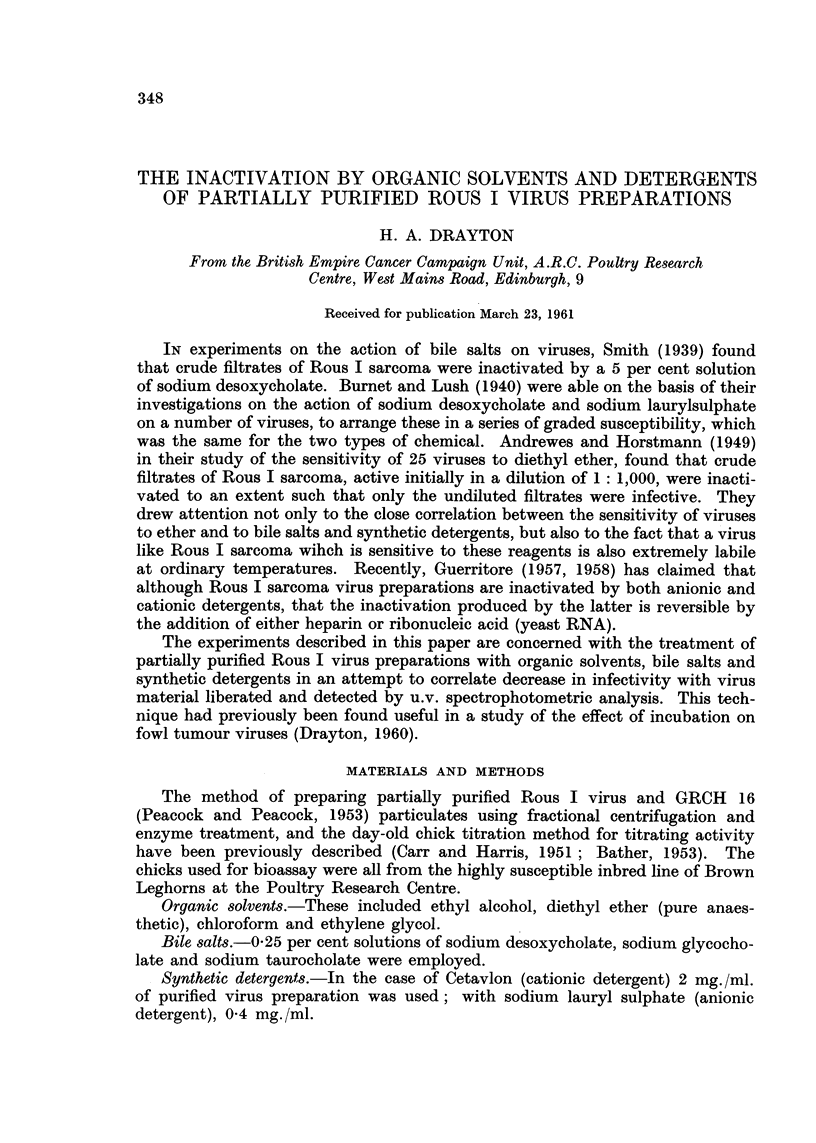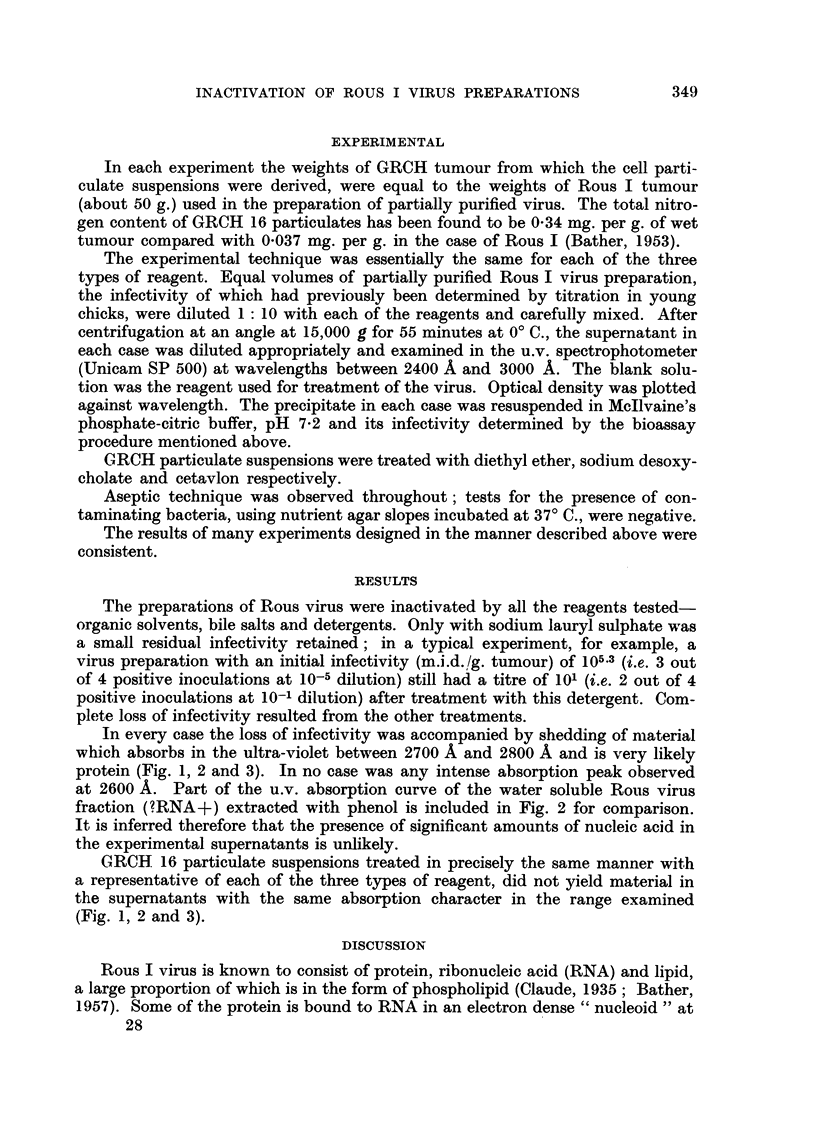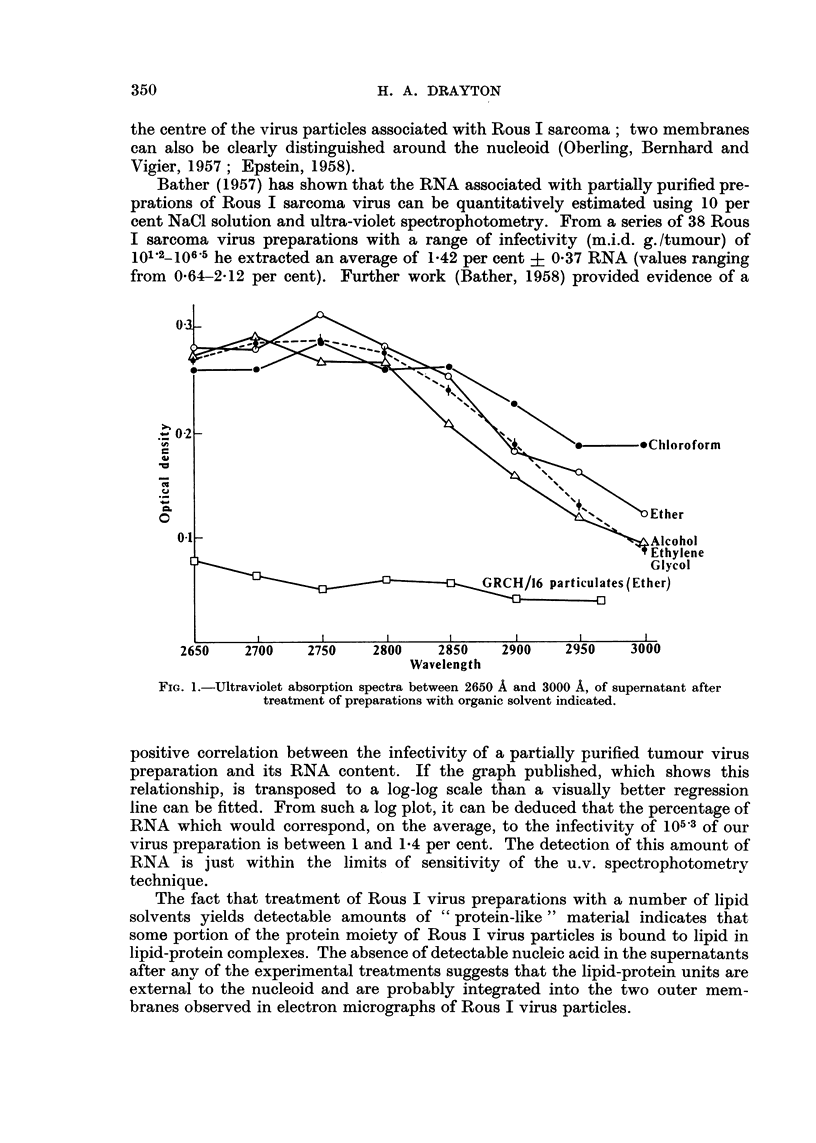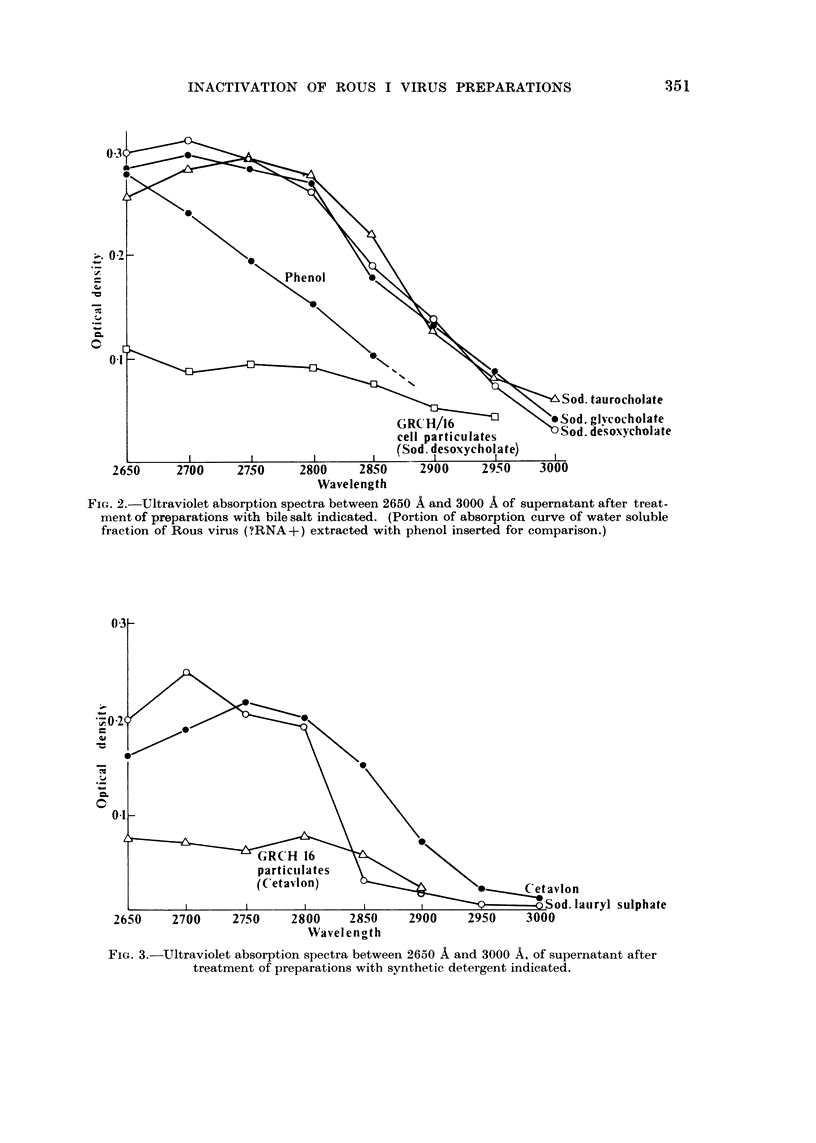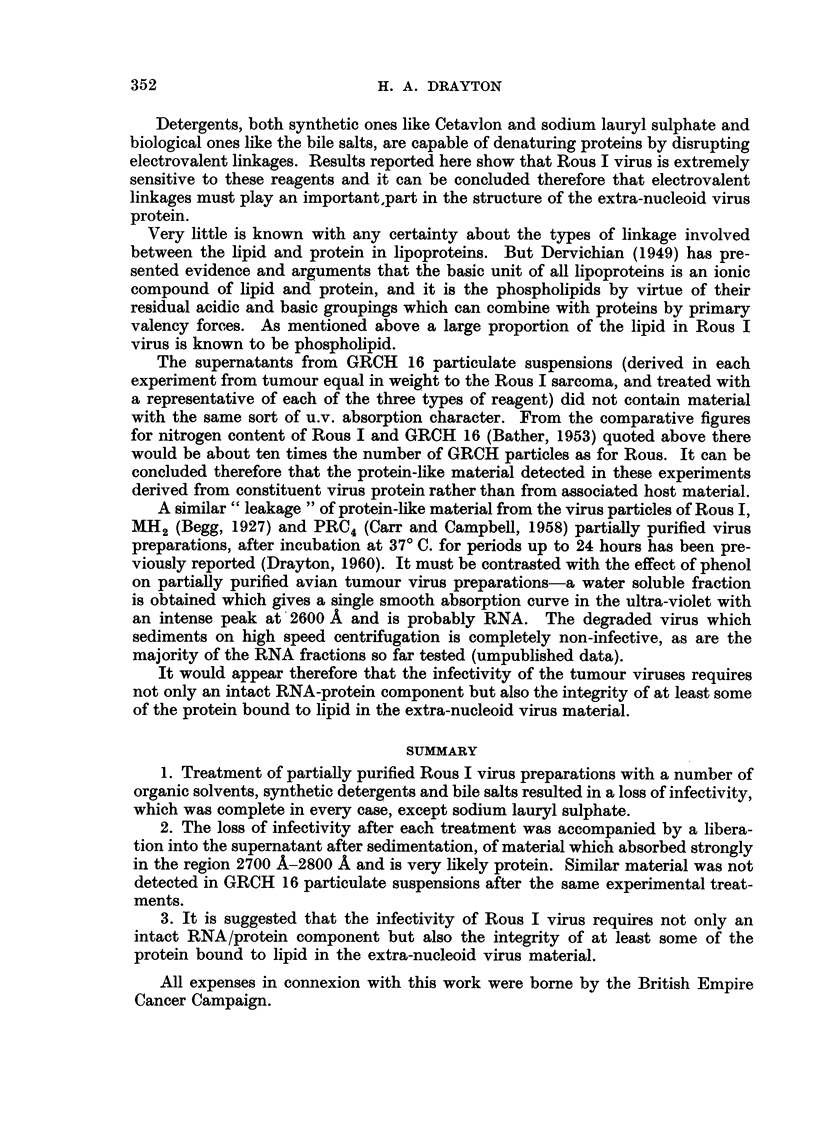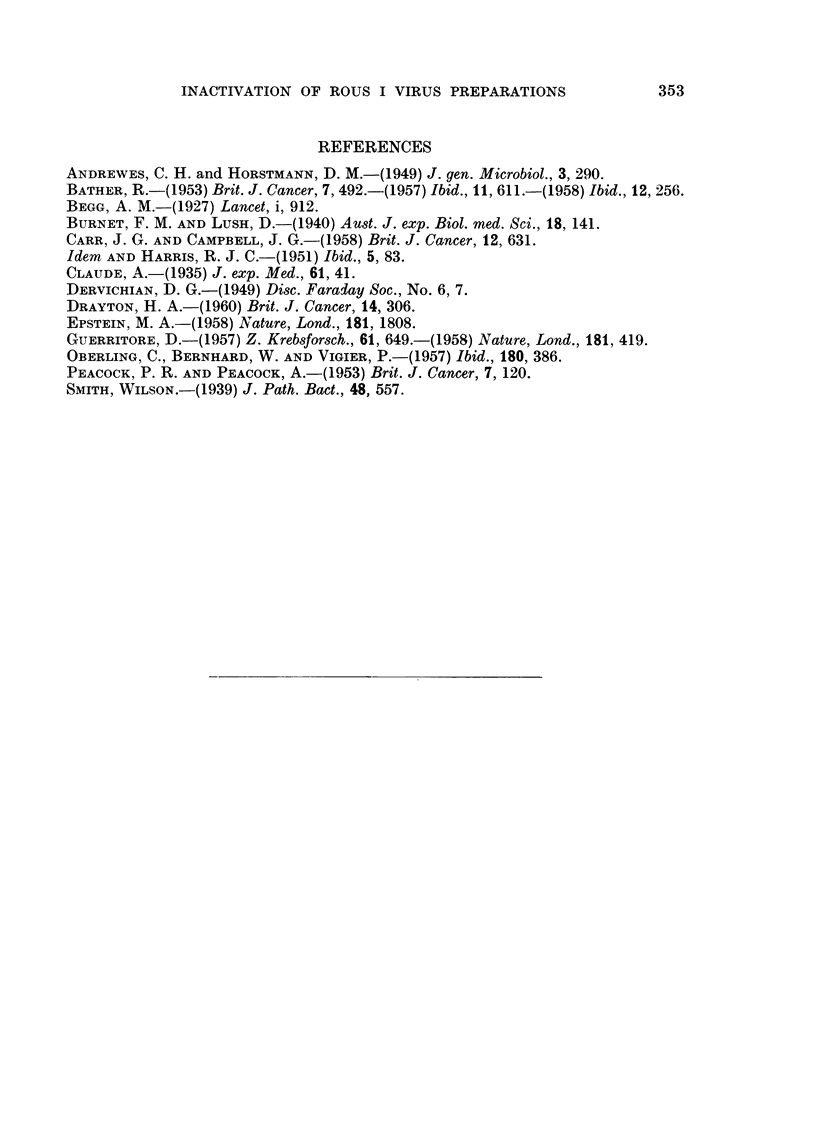# The Inactivation by Organic Solvents and Detergents of Partially Purified Rous I Virus Preparations

**DOI:** 10.1038/bjc.1961.44

**Published:** 1961-06

**Authors:** H. A. Drayton


### THE INACTIVATION BY ORGANIC SOLVENTS AND DETERGENTS OF PARTIALLY PURIFIED ROUS I VIRUS PREPARATIONS

H. A. DRAYTON

*From the British Empire Cancer Campaign Unit, A.R.C. Poultry Research Centre, West Mains Road, Edinburgh, 9*

Received for publication March 23, 1961

IN experiments on the action of bile salts on viruses, Smith (1939) found that crude filtrates of Rous I sarcoma were inactivated by a 5 per cent solution of sodium desoxycholate. Burnet and Lush (1940) were able on the basis of their investigations on the action of sodium desoxycholate and sodium laurylsulphate on a number of viruses, to arrange these in a series of graded susceptibility, which was the same for the two types of chemical. Andrewes and Horstmann (1949) in their study of the sensitivity of 25 viruses to diethyl ether, found that crude filtrates of Rous I sarcoma, active initially in a dilution of 1 : 1,000, were inactivated to an extent such that only the undiluted filtrates were infective. They drew attention not only to the close correlation between the sensitivity of viruses to ether and to bile salts and synthetic detergents, but also to the fact that a virus like Rous I sarcoma wihch is sensitive to these reagents is also extremely labile at ordinary temperatures. Recently, Guerritore (1957, 1958) has claimed that although Rous I sarcoma virus preparations are inactivated by both anionic and cationic detergents, that the inactivation produced by the latter is reversible by the addition of either heparin or ribonucleic acid (yeast RNA).

The experiments described in this paper are concerned with the treatment of partially purified Rous I virus preparations with organic solvents, bile salts and synthetic detergents in an attempt to correlate decrease in infectivity with virus material liberated and detected by u.v. spectrophotometric analysis. This technique had previously been found useful in a study of the effect of incubation on fowl tumour viruses (Drayton, 1960).

### MATERIALS AND METHODS

The method of preparing partially purified Rous I virus and GRCH 16 (Peacock and Peacock, 1953) particulates using fractional centrifugation and enzyme treatment, and the day-old chick titration method for titrating activity have been previously described (Carr and Harris, 1951 ; Bather, 1953). The chicks used for bioassay were all from the highly susceptible inbred line of Brown Leghorns at the Poultry Research Centre.

*Organic solvents.*—These included ethyl alcohol, diethyl ether (pure anaesthetic), chloroform and ethylene glycol.

*Bile salts.*—0·25 per cent solutions of sodium desoxycholate, sodium glycocholate and sodium taurocholate were employed.

*Synthetic detergents.*—In the case of Cetavlon (cationic detergent) 2 mg./ml. of purified virus preparation was used ; with sodium lauryl sulphate (anionic detergent), 0·4 mg./ml.

### EXPERIMENTAL

In each experiment the weights of GRCH tumour from which the cell particulate suspensions were derived, were equal to the weights of Rous I tumour (about 50 g.) used in the preparation of partially purified virus. The total nitrogen content of GRCH 16 particulates has been found to be 0·34 mg. per g. of wet tumour compared with 0·037 mg. per g. in the case of Rous I (Bather, 1953).

The experimental technique was essentially the same for each of the three types of reagent. Equal volumes of partially purified Rous I virus preparation, the infectivity of which had previously been determined by titration in young chicks, were diluted 1 : 10 with each of the reagents and carefully mixed. After centrifugation at an angle at 15,000 *g* for 55 minutes at 0° C., the supernatant in each case was diluted appropriately and examined in the u.v. spectrophotometer (Unicam SP 500) at wavelengths between 2400 Å and 3000 Å. The blank solution was the reagent used for treatment of the virus. Optical density was plotted against wavelength. The precipitate in each case was resuspended in McIlvaine's phosphate-citric buffer, pH 7·2 and its infectivity determined by the bioassay procedure mentioned above.

GRCH particulate suspensions were treated with diethyl ether, sodium desoxycholate and cetavlon respectively.

Aseptic technique was observed throughout ; tests for the presence of contaminating bacteria, using nutrient agar slopes incubated at 37° C., were negative.

The results of many experiments designed in the manner described above were consistent.

### RESULTS

The preparations of Rous virus were inactivated by all the reagents tested—organic solvents, bile salts and detergents. Only with sodium lauryl sulphate was a small residual infectivity retained ; in a typical experiment, for example, a virus preparation with an initial infectivity (m.i.d./g. tumour) of $10^{5 \cdot 3}$ (*i.e.* 3 out of 4 positive inoculations at $10^{-5}$ dilution) still had a titre of $10^{1}$ (*i.e.* 2 out of 4 positive inoculations at $10^{-1}$ dilution) after treatment with this detergent. Complete loss of infectivity resulted from the other treatments.

In every case the loss of infectivity was accompanied by shedding of material which absorbs in the ultra-violet between 2700 Å and 2800 Å and is very likely protein (Fig. 1, 2 and 3). In no case was any intense absorption peak observed at 2600 Å. Part of the u.v. absorption curve of the water soluble Rous virus fraction (?RNA+) extracted with phenol is included in Fig. 2 for comparison. It is inferred therefore that the presence of significant amounts of nucleic acid in the experimental supernatants is unlikely.

GRCH 16 particulate suspensions treated in precisely the same manner with a representative of each of the three types of reagent, did not yield material in the supernatants with the same absorption character in the range examined (Fig. 1, 2 and 3).

### DISCUSSION

Rous I virus is known to consist of protein, ribonucleic acid (RNA) and lipid, a large proportion of which is in the form of phospholipid (Claude, 1935 ; Bather, 1957). Some of the protein is bound to RNA in an electron dense " nucleoid " at the centre of the virus particles associated with Rous I sarcoma ; two membranes can also be clearly distinguished around the nucleoid (Oberling, Bernhard and Vigier, 1957 ; Epstein, 1958).

Bather (1957) has shown that the RNA associated with partially purified preparations of Rous I sarcoma virus can be quantitatively estimated using 10 per cent NaCl solution and ultra-violet spectrophotometry. From a series of 38 Rous I sarcoma virus preparations with a range of infectivity (m.i.d. g./tumour) of $10^{1\cdot2}$–$10^{6\cdot5}$ he extracted an average of 1·42 per cent $\pm$ 0·37 RNA (values ranging from 0·64–2·12 per cent). Further work (Bather, 1958) provided evidence of a

FIG. 1.—Ultraviolet absorption spectra between 2650 Å and 3000 Å, of supernatant after treatment of preparations with organic solvent indicated.

positive correlation between the infectivity of a partially purified tumour virus preparation and its RNA content. If the graph published, which shows this relationship, is transposed to a log-log scale than a visually better regression line can be fitted. From such a log plot, it can be deduced that the percentage of RNA which would correspond, on the average, to the infectivity of $10^{5\cdot3}$ of our virus preparation is between 1 and 1·4 per cent. The detection of this amount of RNA is just within the limits of sensitivity of the u.v. spectrophotometry technique.

The fact that treatment of Rous I virus preparations with a number of lipid solvents yields detectable amounts of "protein-like" material indicates that some portion of the protein moiety of Rous I virus particles is bound to lipid in lipid-protein complexes. The absence of detectable nucleic acid in the supernatants after any of the experimental treatments suggests that the lipid-protein units are external to the nucleoid and are probably integrated into the two outer membranes observed in electron micrographs of Rous I virus particles.

Fig. 2.—Ultraviolet absorption spectra between 2650 Å and 3000 Å of supernatant after treatment of preparations with bile salt indicated. (Portion of absorption curve of water soluble fraction of Rous virus (?RNA+) extracted with phenol inserted for comparison.)

Fig. 3.—Ultraviolet absorption spectra between 2650 Å and 3000 Å, of supernatant after treatment of preparations with synthetic detergent indicated.

Detergents, both synthetic ones like Cetavlon and sodium lauryl sulphate and biological ones like the bile salts, are capable of denaturing proteins by disrupting electrovalent linkages. Results reported here show that Rous I virus is extremely sensitive to these reagents and it can be concluded therefore that electrovalent linkages must play an important,part in the structure of the extra-nucleoid virus protein.

Very little is known with any certainty about the types of linkage involved between the lipid and protein in lipoproteins. But Dervichian (1949) has presented evidence and arguments that the basic unit of all lipoproteins is an ionic compound of lipid and protein, and it is the phospholipids by virtue of their residual acidic and basic groupings which can combine with proteins by primary valency forces. As mentioned above a large proportion of the lipid in Rous I virus is known to be phospholipid.

The supernatants from GRCH 16 particulate suspensions (derived in each experiment from tumour equal in weight to the Rous I sarcoma, and treated with a representative of each of the three types of reagent) did not contain material with the same sort of u.v. absorption character. From the comparative figures for nitrogen content of Rous I and GRCH 16 (Bather, 1953) quoted above there would be about ten times the number of GRCH particles as for Rous. It can be concluded therefore that the protein-like material detected in these experiments derived from constituent virus protein rather than from associated host material.

A similar " leakage " of protein-like material from the virus particles of Rous I, $MH_2$ (Begg, 1927) and $PRC_4$ (Carr and Campbell, 1958) partially purified virus preparations, after incubation at 37° C. for periods up to 24 hours has been previously reported (Drayton, 1960). It must be contrasted with the effect of phenol on partially purified avian tumour virus preparations—a water soluble fraction is obtained which gives a single smooth absorption curve in the ultra-violet with an intense peak at 2600 Å and is probably RNA. The degraded virus which sediments on high speed centrifugation is completely non-infective, as are the majority of the RNA fractions so far tested (umpublished data).

It would appear therefore that the infectivity of the tumour viruses requires not only an intact RNA-protein component but also the integrity of at least some of the protein bound to lipid in the extra-nucleoid virus material.

### SUMMARY

1. Treatment of partially purified Rous I virus preparations with a number of organic solvents, synthetic detergents and bile salts resulted in a loss of infectivity, which was complete in every case, except sodium lauryl sulphate.

2. The loss of infectivity after each treatment was accompanied by a liberation into the supernatant after sedimentation, of material which absorbed strongly in the region 2700 Å–2800 Å and is very likely protein. Similar material was not detected in GRCH 16 particulate suspensions after the same experimental treatments.

3. It is suggested that the infectivity of Rous I virus requires not only an intact RNA/protein component but also the integrity of at least some of the protein bound to lipid in the extra-nucleoid virus material.

All expenses in connexion with this work were borne by the British Empire Cancer Campaign.

### REFERENCES

Andrewes, C. H. and Horstmann, D. M.—(1949) *J. gen. Microbiol.*, **3**, 290.
Bather, R.—(1953) *Brit. J. Cancer*, **7**, 492.—(1957) *Ibid.*, **11**, 611.—(1958) *Ibid.*, **12**, 256.
Begg, A. M.—(1927) *Lancet*, i, 912.
Burnet, F. M. and Lush, D.—(1940) *Aust. J. exp. Biol. med. Sci.*, **18**, 141.
Carr, J. G. and Campbell, J. G.—(1958) *Brit. J. Cancer*, **12**, 631.
*Idem* and Harris, R. J. C.—(1951) *Ibid.*, **5**, 83.
Claude, A.—(1935) *J. exp. Med.*, **61**, 41.
Dervichian, D. G.—(1949) *Disc. Faraday Soc.*, No. 6, 7.
Drayton, H. A.—(1960) *Brit. J. Cancer*, **14**, 306.
Epstein, M. A.—(1958) *Nature, Lond.*, **181**, 1808.
Guerritore, D.—(1957) *Z. Krebsforsch.*, **61**, 649.—(1958) *Nature, Lond.*, **181**, 419.
Oberling, C., Bernhard, W. and Vigier, P.—(1957) *Ibid.*, **180**, 386.
Peacock, P. R. and Peacock, A.—(1953) *Brit. J. Cancer*, **7**, 120.
Smith, Wilson.—(1939) *J. Path. Bact.*, **48**, 557.